# Dietary pattern and leisure time activity of overweight and normal weight children in Germany: sex-specific differences

**DOI:** 10.1186/1475-2891-12-14

**Published:** 2013-01-15

**Authors:** Ina B Maier, Yelda Özel, Sabine Wagnerberger, Stephan C Bischoff, Ina Bergheim

**Affiliations:** 1 Department of Nutritional Medicine (180a), University of Hohenheim, Fruwirthstrasse 12, Stuttgart, 70599, Germany

**Keywords:** Children, Dietary pattern, Obesity, Overweight, Sex, Physical activity

## Abstract

**Background:**

Several studies indicate that dietary pattern and leisure time activities of adults not only differ between sexes but also between overweight and normal weight individuals. The aim of the present study was to determine if sex-specific differences in dietary pattern and leisure time activity already exist and are associated with weight status in young childhood.

**Methods:**

Nutritional intake, anthropometric parameters, leisure time activities and socio- demographical factors were assessed in 100 overweight and 51 normal weight children (81 girls and 70 boys), aged 5–8 years.

**Results:**

In general, independent of body weight, boys ate more cheese while girls consumed more vegetables and spent more time with sedentary activities. Moreover, regardless of sex, total energy and macronutrient intake did not differ between normal weight and overweight children. Also, time spent with sportive activities did not differ between groups; however, overweight boys spent significantly more leisure time with sedentary activities than normal weight boys. Furthermore, BMI of mothers and time spent with sedentary activities were identified as independent risk factors for the development of overweight when performing multiple regression analyses.

**Conclusions:**

Taken together, results of our study suggest that already at young age sex influences dietary pattern independent of body weight. Furthermore, an increased time spent with sedentary activities and an elevated maternal BMI were found to be associated with an elevated body weight in children. (Trial registration: NCT01306396)

## Introduction

The prevalence of overweight and obesity among children and adolescents in industrialized countries has increased dramatically throughout the last three decades. In Germany, the German Health Interview and Examination Survey for Children and Adolescents (KiGGS) recently revealed that ~15% of children and adolescents are overweight and of these ~6.3% are even obese according to the national reference [1]. Overweight is often described as the result of an imbalance between energy intake and energy expenditure [2]. Results of several studies indicate that the dietary pattern of normal weight and overweight children and adolescents differ and that overweight children more often consume food considered as unhealthy [3]. Indeed, in these studies it was shown that overweight children and adolescents ate sugar and snacks more frequently than normal weight children and adolescents, whereas legumes, vegetables and fruits were consumed less frequently. Contrary to these findings, a study by Janssen et al. found no clear association between dietary habits and the prevalence of overweight in adolescents in Canada [4]. Furthermore, Janssen et al. [5] who analysed dietary pattern of 10–16 year old adolescents assessed in 34 different, mainly European countries, reported that the consumption of fruits, vegetables, sweets and soft drinks differs regionally.

Another factor often discussed to influence dietary pattern is sex. Results of several studies in adults indicate that women consume more fruit and vegetable than men whereas men eat more meat, eggs, milk and sweets [6,7]. Results of epidemiological studies suggest that dietary habits are acquired in childhood and are often maintained into adulthood [8]. In line with this hypothesis, several studies analyzing dietary pattern in older children, adolescents and young adults found that fruit and vegetable intake of girls was markedly higher in comparison to boys whereas boys ate more milk products, cereals and meat [9]. However, only scarce information on dietary pattern and possible sex-specific differences of children below the age of 10 years is available and results vary between countries [5,10]. Starting from this background the aim of the present study was to determine if sex-specific differences in dietary pattern and leisure time activity already exist in boys and girls aged 5–8 years and if these differ between normal weight and overweight children.

### Methods and procedures

#### Subjects

The study was approved by the ethics committee of the “Landesärztekammer Baden-Württemberg” (Stuttgart, Germany) and was performed in accordance with the ethical standards of the Helsinki Declaration from 1975 and revised in 1983. Written informed consent was obtained from all subjects and their parents or guardians. A total of 100 overweight children (43 boys, 57 girls) and 51 normal weight children (27 boys, 24 girls) aged 5–8 years were included in the study. All participants were recruited between April 2009 and December 2010 through 117 primary schools in Stuttgart (Germany). The study is registered at http://www.clinicaltrials.gov (NCT01306396).

#### Anthropometric measurements

Weight was measured without shoes in light clothing to the nearest 0.1 kg by using an electronic calibrated scale (RIBA Model ABW-150K, Geldermalsen, Netherlands). Height was measured in a standing position without shoes using a portable height measure (Leicester Height Measure, Invicta, Leicester, England) to the nearest 0.1 cm. BMI (Body Mass Index) was calculated as weight in kilograms divided by height in meters squared and was expressed in kg/m^2^. In accordance with the German association of obesity in childhood and adolescence (AGA) BMI values were used to classify children as normal weight (BMI < 90th percentile BMI), as overweight (BMI > 90th percentile BMI) or obese (BMI > 97th percentile BMI) [11]. In addition, BMI-SDS (BMI standard deviation score) was also calculated according to the German association of obesity in childhood and adolescence using reference data for German children [11].

#### Dietary assessment

To assess dietary pattern as well as total intake of energy and macronutrients, two 24-h dietary recalls were performed on independent days with each study participant in the presence of their respective guardian by an experienced dietician. To assess meal sizes, children and parents were shown pictures of meals in different sizes (EPIC picture book, source: International agency for research on cancer). Nutritional data were analysed using the computer software EBISpro (Version 8.0, 2007, Germany). To investigate sex-specific differences in dietary pattern, intake of the following 12 different food groups was compared between all boys and girls: fruits, vegetables, meat and sausages, fish, pasta and rice and potato, bread and cereals, fat, cheese, milk, eggs, sweets, others. To check for underreporting, the ratio of reported total energy intake (TEI) and predicted individual basal metabolic rate (BMR) was used as described previously by others [12]. As described before by Sichert-Hellert et al. [13] only recalls with a ratio above the age- and sex-specific cut-off values (for boys 1.04 and girls 1.01) were included in the analysis. As a result, 6 overweight children had to be excluded from the analysis of dietary intake.

#### Leisure time activities and socio-demographic data

Leisure time activities were assessed using a questionnaire adapted and modified from KiGGS [14]. In addition to the general assessment of time spent physical active or sedentary, the questionnaire assessed the kind of physical activity (e.g. cycling or time spent with organized sportive activities like gymnastics and soccer) and sedentary activities (e.g. reading, painting, watching TV). Questions were imposed to both parents/caregivers and children, respectively. Furthermore, socio-demographic characteristics like age, sex, ethnicity, socio-economic status, assessed by the mothers’ and fathers’ level of education, and the weight status of the parents were recorded.

#### Statistical analysis

Results are described as means ± SD. Normality of data was checked using Kolmogorov-Smirnov-Test. Data were analyzed using the *χ*^2^ test and the Mann–Whitney *U* test. The relationship between weight status (BMI-SDS) and potentially risk factors for overweight (e.g. BMI of mothers and fathers, energy intake, sedentary activities) were assessed using uni- and multivariate linear regression analyses. Only variables that were significant in the univariate analyses were included in the multivariate model. P-values <0.05 were defined as the level of significance using the software SPSS statistics (Version 17.0, 2008, SPSS Inc., Chicago, IL, USA).

## Results

### Socio-demographical and anthropometric characteristics

Socio-demographical and anthropometric characteristics of study participants are summarized in Table 1. When comparing all children regardless of body weight, no differences between sexes were found regarding age, ethnicity, socio-economic status and anthropometric parameters (e.g. BMI and BMI-SDS). Socio-demographic parameters of normal weight boys and girls did not differ; however, normal weight girls were significantly taller and heavier than normal weight boys, but mean BMI-SDS did not differ between sexes. BMI-SDS was significantly higher in overweight boys than girls. Overweight children regardless of sex were significantly taller, heavier and had a higher BMI-SDS than the corresponding normal weight children (Table 1). Age, ethnicity and socio-economic status did not differ between overweight and normal weight children (Table 1). Average BMI of mothers of overweight children was significantly higher in comparison to that of normal weight children. A similar difference was not found for the BMIs of fathers. In ~75% of all families mothers were described to be the primary attachment figure (see Table 1).

**Table 1:** Socio-demographical and anthropometric characteristics of study participants

	**All**		**Girls**			**Boys**		
	**OW**	**NW**	**All**	**OW**	**NW**	**All**	**OW**	**NW**
**n**	**94**	**51**	**78**	**54**	**24**	**67**	**40**	**27**
Age (years)	7.6 ± 1.1	7.3 ± 1.1	7.5 ± 1.1	7.5 ± 1.1	7.5 ± 1.2	7.5 ± 1.0	7.7 ± 1.0	7.2 ± 0.9^c^
Ethnicity (%)								
caucasian	70	73	68	69	67	75	73	77.8
asiatic	30	25	31	31	29	25	27	22.2
black	-	2	1	-	4	-	-	-
Graduation mothers (%)								
< 10 years	28	20	24	26	21	25	30	18
= 10 years	23	35	33	30	42	21	15	30
> 10 years	49	45	42	44	37	54	55	52
Graduation fathers (%)								
< 10 years	31	31	29	26	38	33	38	26
= 10 years	25	22	21	26	8	28	25	33
> 10 years	44	47	50	48	54	39	37	41
Primary attachment figure (%)								
Mother	76	75	77	74	83.3	74	79	67
Father	2	6	4	4	4.2	3	0	8
Others	22	19	19	22	12.5	23	21	25
Mothers’ BMI (kg/m^2^)	27.6 ± 6.5	23.6 ± 4.0^a^	26.6 ± 6.1	27.3 ± 6.6	24.9 ± 4.7	25.7 ± 6.0	27.9 ± 6.6	22.5 ± 2.8^c^
Fathers’ BMI (kg/m^2^)	28.8 ± 4.9	27.3 ± 3.8	28.3 ± 4.8	29.0 ± 5.0	26.9 ± 3.9	28.2 ± 4.4	28.5 ± 4.8	27.7 ± 3.8
Weight (kg)	36.1 ± 7.2	26.7 ± 3.9^a^	33.0 ± 7.2	35.3 ± 7.1	27.9 ± 4.2^b^	32.5 ± 8.3	37.1 ± 7.4	25.5 ± 3.4^c,d^
Height (m)	1.30 ± 0.1	1.26 ± 0.07^a^	1.29 ± 0.09	1.29 ± 0.1	1.28 ± 0.07	1.28 ± 0.09	1.31 ± 0.09	1.24 ± 0.07^c,d^
BMI (kg/m^2^)	21.2 ± 2.1	16.8 ± 1.2^a^	19.8 ± 2.6	21.0 ± 2.1	17.0 ± 1.4^b^	19.4 ± 3.0	21.4 ± 2.1	16.5 ± 1.0^c^
BMI-SDS	1.89 ± 0.47	0.44 ± 0.58^a^	1.41 ± 0.81	1.81 ± 0.47	0.50 ± 0.67^b^	1.35 ± 0.93	2.00 ± 0.46^b^	0.38 ± 0.48^c^

Values are means ± SD. ^a^p < 0.05 in comparison to all OW children. ^b^p < 0.05 in comparison to OW girls. ^c^p < 0.05 in comparison to OW boys. ^d^p < 0.05 in comparison to NW girls. *OW*, overweight, *NW*, normal weight.

### Differences in nutrient intake

When comparing nutritional intake of normal weight and overweight children no differences in energy and macronutrient intake were found (Table 2). Interestingly, mean energy intake as well as total carbohydrate, protein and fat intake of all boys was significantly higher in comparison to all girls regardless of weight status. Furthermore, total protein intake was also significantly higher in overweight boys compared to overweight girls. In normal weight boys, a significantly higher intake of carbohydrates was found when compared to normal weight girls (Table 2).

**Table 2:** Nutritional intake and leisure time activities of study participants

	**All**		**Girls**			**Boys**		
	**OW**	**NW**	**All**	**OW**	**NW**	**All**	**OW**	**NW**
**n**	**94**	**51**	**78**	**54**	**24**	**67**	**40**	**27**
Energy (kcal/d)	1876 ± 418	1934 ± 490	1792 ± 366	1786 ± 330	1806 ± 445	2017 ± 495^a^	1997 ± 492	2048 ± 507
Carbohydrate (g/d)	231 ± 57	248 ± 66	225 ± 50	223 ± 49	228 ± 52	252 ± 68^a^	243 ± 64	265 ± 72^b^
Protein (g/d)	62.7 ± 17.1	59.4 ± 19.3	57.7 ± 16.5	59.1 ± 15.6	54.4 ± 18.2	66.0 ± 18.6^a^	67.5 ± 18.1^c^	63.8 ± 19.5
Fat (g/d)	77.0 ± 23.1	77.4 ± 26.6	72.4 ± 21.2	71.8 ± 18.7	73.9 ± 26.6	82.6 ± 26.6^a^	84.0 ± 26.7	80.6 ± 26.8
Sportive activities (h/week)	14.1 ± 7.8	14.3 ± 7.1	14.2 ± 7.9	14.2 ± 8.0	14.1 ± 7.7	14.2 ± 7.2	14.0 ± 7.7	14.5 ± 6.7
Sedentary activities (h/week)	22.4 ± 12.0	17.0 ± 10.5^d^	22.2 ± 11.8	23.4 ± 11.8	19.4 ± 11.5	18.6 ± 11.5^a^	21.2 ± 12.3	14.8 ± 9.1^e^

Values are means ± SD. ^a^p < 0.05 in comparison to all girls. ^b^p < 0.05 in comparison to normal weight girls. ^c^p < 0.05 in comparison to OW girls. ^d^p < 0.05 in comparison to all OW children. ^e^p < 0.05 in comparison to OW boys. *OW*, overweight, *NW*, normal weight.

### Differences in dietary pattern

**Figure 1:**
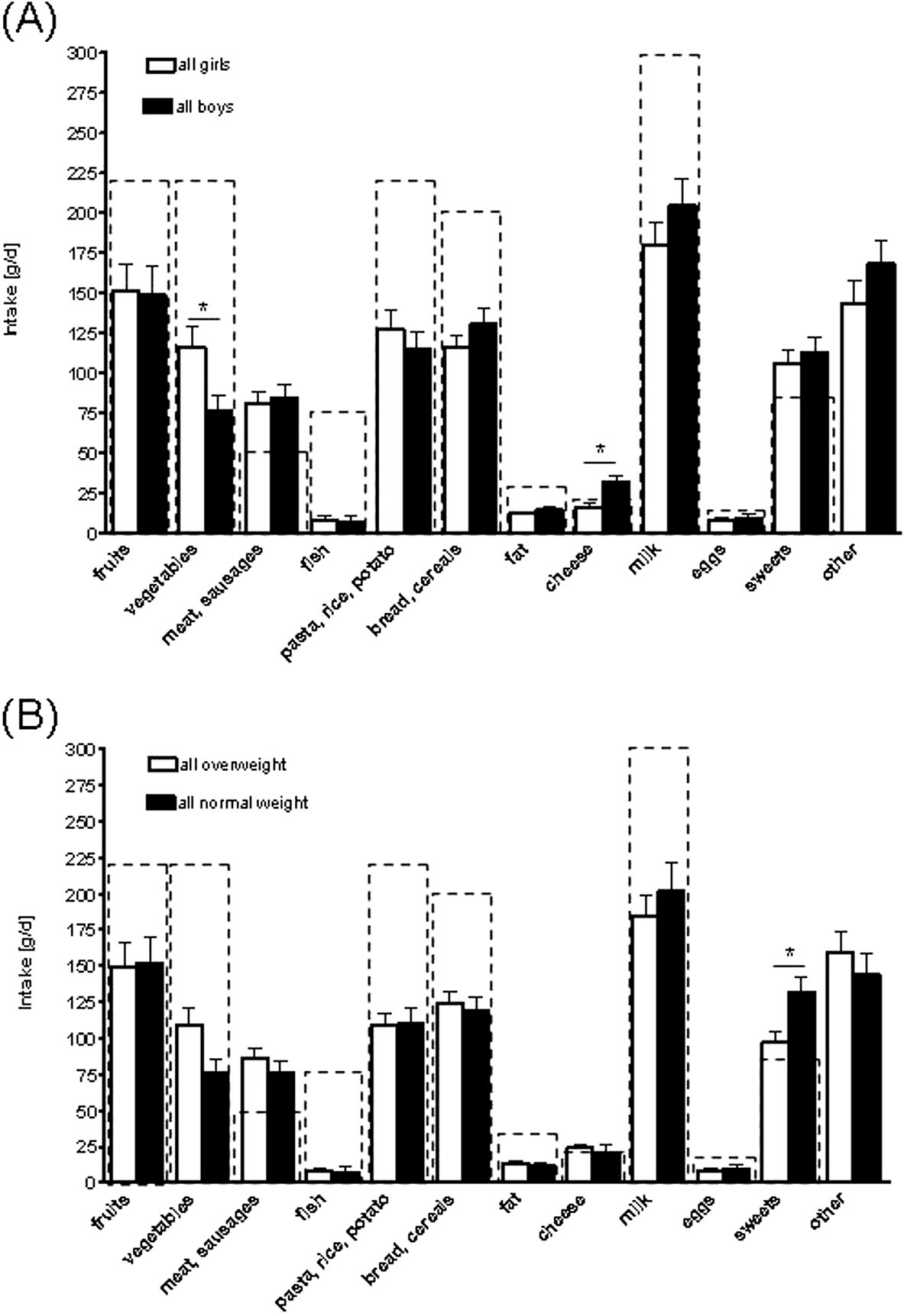
**Intake of different food groups of (A) all boys and girls and of (B) all overweight and normal weight children.** Recommendations of the German research institute for nutrition of children (FKE) are shown as dashed lines [15]. *p < 0.05.

In general, in overweight and normal weight children, intake of different food groups e.g. vegetables and fruits as well as meat did not meet the recommendations of the German research institute for nutrition of children (FKE) [15] (see Figure 1). In Figure 1 dietary pattern of study participants are summarized. Boys ate significantly more cheese in comparison to the girls whereas in girls vegetable consumption was significantly higher. Similar differences were also found when overweight children were only analysed; however, in normal weight children vegetable intake did not differ between sexes (see Additional file 1).

When comparing dietary pattern of all overweight and normal weight children (Figure 1B) a significantly higher intake of sweets was found for normal weight children. Similar results were also found when dietary pattern of normal weight and overweight boys were compared (see Additional file 1).

### Leisure time activities

In Table 2 leisure time activities are summarized for all study participants. When comparing girls and boys independent of their weight status, time of physical activity during leisure time was similar (~14.5 h/week). However, girls reported to spend significantly more time with sedentary activities (e.g. painting, reading) during their leisure time than boys. A similar trend was also found when comparing normal weight girls and boys (p = 0.069) but not for overweight children. When comparing average leisure time activities of all overweight and normal weight children, overweight children were found to spend significantly more time with sedentary activities in their leisure time. Similar results were also found for overweight and normal weight boys but not for overweight and normal weight girls.

When performing a regression analysis for BMI of mothers, BMI of fathers and sedentary activities, respectively a relation of these parameters with BMI-SDS of children was found (Table 3). However, when performing a stepwise multiple linear regression analysis, only BMI of mothers (beta = 0.379, p < 0.05) and time spent with sedentary activities (beta = 0.213, p < 0.05) were found to be independent risk factors for BMI-SDS.

**Table 3:** Results of univariate regression analysis for risk factors for overweight using BMI-SDS as dependent variable

**Independent variable**	**Regression coefficient**	**p-value**
BMI mothers	0,158	0,001
BMI fathers	0,03	0,05
Energy intake	−0,004	0,527
Sedentary activities	0,072	0,001
Sportive activities	−0,007	0,997

## Discussion

World-wide the prevalence of overweight and obesity has increased dramatically during the last three decades not only in adults but also in children. Intense research efforts have been undertaken to clarify mechanisms involved in the development of overweight and obesity in humans; however, despite these efforts, mechanisms involved are still poorly understood and universally accepted therapies are still lacking. Results of several epidemiologic studies indicated that dietary pattern of adults not only differ markedly between sexes [10,16], but also between overweight and normal weight individuals [17]. However, similar studies have rarely been performed in children under the age of 10 years and results are contradictory [4,5]. In the present study, we tested the hypothesis that dietary intake differs between normal and overweight children and between boys and girls. It was shown before by others that a long-term overnutrition of ~100 kcal/d may cause overweight [18]. In the present study, total energy and macronutrient intake did not differ between normal weight and overweight children and all children being overweight or even obese, reached the recommendation to spend one hour per day with sportive activities regardless of weight status [19]. However, leisure time spent with sedentary activity like watching TV was markedly longer in overweight children regardless of sex whereas time spent physical active (e.g. playing soccer) did not differ between groups regardless of sex, suggesting that nutritional intake (e.g. total energy intake) of overweight children may have been too high in comparison to energy expenditure. Indeed in the present study, sedentary activity was identified as an independent risk factor for the development of overweight in children. These findings are in line with those of other groups, who found a positive association between screen time or total media consumption and the degree of overweight [5]. In addition, results of a meta-analysis suggest that media consumption often replaces physical activity (e.g. sport) during leisure time [20]. Contrary to the findings in adults [17], in the present study, intake of sweets was markedly higher in normal weight children than in overweight or obese children. Still, intake of different food groups e.g. vegetables and fruits as well as meat intake of children in general did not meet the recommendations of the German research institute for nutrition of children (FKE). These findings are in line with those of others. Indeed, results of a meta-analysis of studies performed in 34 different countries revealed that normal weight children quite frequently eat more sweets than overweight children [5]. Furthermore, Ortega et al. also reported that total energy intake between overweight and normal weight adolescents did not differ [21], whereas in the studies of Hassapidou et al. [3] and Rocandio et al. [2], total energy intake of normal weight adolescents was even higher than that of overweight study participants. In line with our findings, in the study of Kersting et al. it was also found that vegetable and meat intake of German children do not meet the recommendations of the German research institute for nutrition of children (FKE) [22].

Interestingly, whereas BMI of fathers did not differ between normal weight and overweight children, mean BMI of mothers of overweight children was markedly higher than that of the normal weight children. This difference was even more pronounced in boys than in girls, which might be explained with the markedly smaller difference in BMI-SDS between overweight and normal weight girls (Δ boys: 1.62 vs. Δ girls: 1.31). Furthermore, multiple regression analysis revealed BMI of mothers as independent risk factor for elevated BMI-SDS of children. Danielzik et al. also found a closer association between children’s BMI and maternal BMI than paternal BMI [23]. The association with maternal overweight may have different reasons and may even be multifactorial. Indeed, results of studies performed in twins suggest that genetic factors may have a strong effect on the variation of BMI [24]. In addition, parents and herein particularly mothers’ behaviour may also play an important role, because children’s food environment is usually influenced more by mothers than by fathers [25]. Furthermore, the results of Fogelholm et al. indicated that physical activity pattern of children are also associated with that of parents [26]. In the present study ~75% of study participants claimed their mothers to be the primary attachment figure. In addition, the results of the report of the German association of nutrition released in 2008 indicates that only 17% of students, aged 14 years eat lunch at school, indicating that lunch at home is still important in Germany [27]. However, as neither physical activity nor dietary pattern and nutritional intake or genetic polymorphisms of parents were assessed in the present study, possible causes of the positive association of maternal BMI with that of children remains to be determined. Taken together, results of the present study suggest that neither a marked overnutrition nor an “abnormal” dietary pattern are responsible for the elevated body weight in overweight children, but rather leisure time activity as well as maternal BMI may be risk factors for the development of obesity. However, reasons for this altered physical activity pattern (e.g. negative parental model, genetic backgrounds) but also the maternal influence on children’ s body weight remain to be determined.

### Sex-specific differences in dietary pattern

Sex-specific differences in nutritional intake and dietary pattern have frequently been reported for adults but also for adolescents (for overview see [6,7,28]). In the present study, we found that boys, regardless of BMI, consumed markedly more cheese while girls, in general, ate more vegetables. In adults, sex-specific differences in dietary pattern and particularly the higher intake of fruit and vegetable were claimed to be a result of the higher interest of women in nutrition [29] and their better knowledge of the relation of eating pattern and disease prevention [6]. However, during childhood parents and herein particularly mothers have a prominent responsibility for children’s diet and it seems to be rather unlikely that parents alter dietary pattern in consideration of sex [30]. Causes of these sex-specific differences in dietary pattern therefore remain to be determined. As already described in other studies particularly for adolescents and adults [16,31], we found that total energy and macronutrient intake in boys was markedly higher than in girls, despite only slight differences in weight status. One explanation for this could be the differences in leisure time activities, as girls spent significantly more time with sedentary activities than boys. This is in line with the findings of Riddoch et al. who reported sex-specific differences in sportive activities [32]. Furthermore, several studies demonstrated a lower fat mass and higher bone-free lean tissue mass in boys already before the onset of puberty [33], which may result in an increased energy expenditure. However, exact mechanisms underlying these sex-specific differences have to be determined. Taken together, the results of the present study suggest that dietary pattern differs between boys and girls, aged 5–8 years, regardless of body weight status. Furthermore, the results also indicate that sex-specific differences in leisure time activities are responsible for the fact that no differences in body weight status were found between boys and girls, despite the higher total energy and macronutrient intake in boys.

### Limitations

Our study has some limitations that need to be considered when interpreting the data. First, nutritional intake was self-reported and might be influenced by recording errors or under-reporting. This is a problem often described in the context of nutritional interviews, especially when performed in overweight adults and children [20,34]. Additionally, it cannot be ruled out that overweight and obese parents tended to underestimate dietary intake of their children. However, as mentioned before, our findings are in line with those of other larger studies performed in Europe. Second, leisure time activities were also self-reported and not assessed by objective measurements such as accelerometers. Indeed, over-reporting of physical activity is also a problem often found in overweight people [35]. Third, the results are not representative for the whole population, as the study sample consists mainly of Caucasian children and parents with an educational level higher than 10 years and it was a rather small sample. Forth, data of this cross-sectional study only represent a short window in time, whereas the development of obesity is clearly a long-term issue. Furthermore, the possibility of statistical type II error cannot be ruled out.

## Conclusion

In conclusion, results of our study suggest that not a drastic overnutrition is associated with the development of overweight in children. Rather, leisure time activity and maternal body weight, be it through dietary pattern, sedentary activity or genes, may be critical determinants of weight status of children. Furthermore, the results of the present study also suggest that, independent of weight status, dietary pattern differ in a sex-specific manner already during childhood. Therefore, future efforts to prevent childhood obesity should 1) focus on parental education programs, not only regarding nutritional intake but also focusing on decreasing sedentary activity during leisure time (e.g. media consumption) of overweight children and 2) take the sex-specific differences in dietary pattern into account.

## Abbreviations

KiGGS: German health interview and examination survey for children and adolescents; BMI: Body Mass Index; BMI-SDS: BMI standard deviation score.

## Competing interests

The authors declare that they have no competing interests.

## Authors’ contributions

IBM conducted research, analysed data and wrote paper, YÖ carried out nutritional interviews, SW conducted research, SCB have been involved in drafting the manuscript, IB designed research, wrote paper and had primary responsibility for final content. All authors read and approved the final manuscript.

## Supplementary Material

Additional file 1 **Intake of different food groups of (A) normal weight girls and normal weight boys and of (B) overweight girls and overweight boys.** Recommendations of the German research institute for nutrition of children (FKE) are shown as dashed lines [15]. *p < 0.05. Click here for file
