# Supplementary material for: Dietary pattern and leisure time activity of overweight and normal weight children in Germany: sex-specific differences
Source: Nutr J. 2013 Jan 15;12:14. doi: 10.1186/1475-2891-12-14 (PMC3585891; doi:10.1186/1475-2891-12-14)
Supplement: Additional file 1 — Intake of different food groups of (A) normal weight girls and normal weight boys and of (B) overweight girls and overweight boys. Recommendations of the German research institute for nutrition of children (FKE) are shown as dashed lines [15]. *p < 0.05. [file 1475-2891-12-14-S1.pdf]

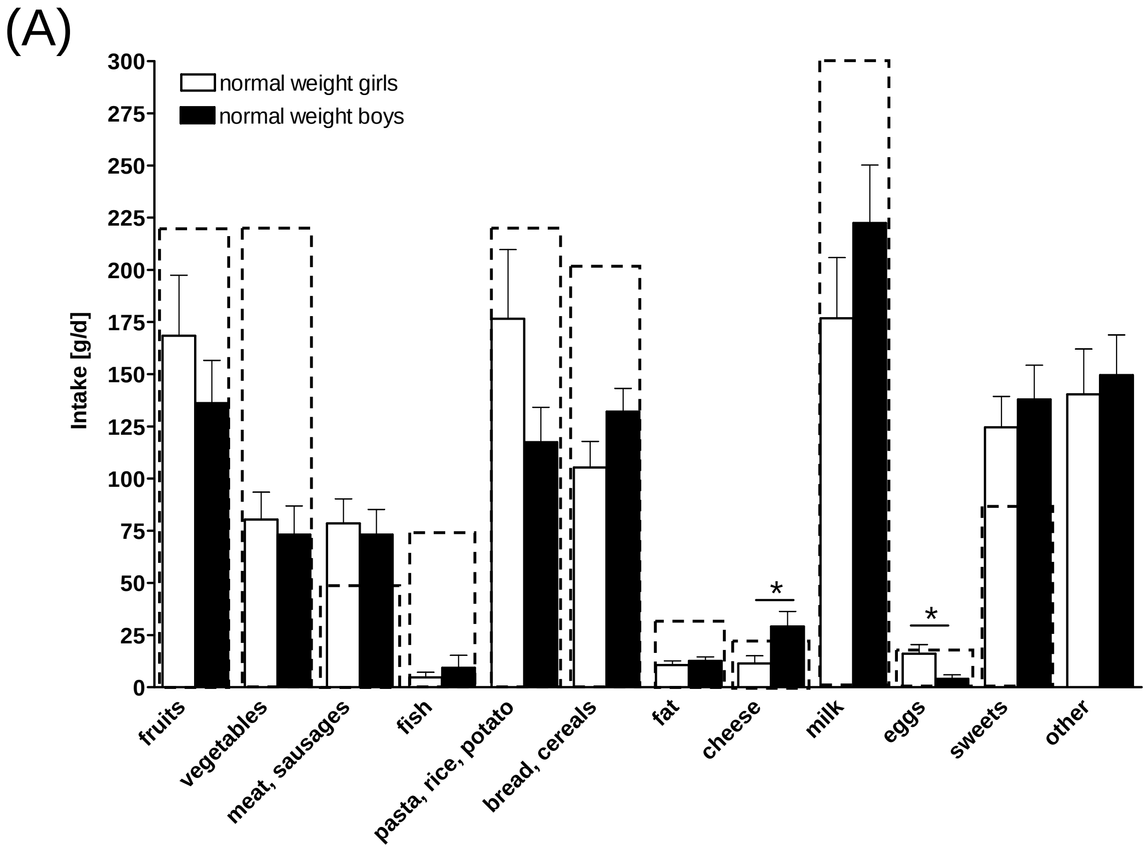

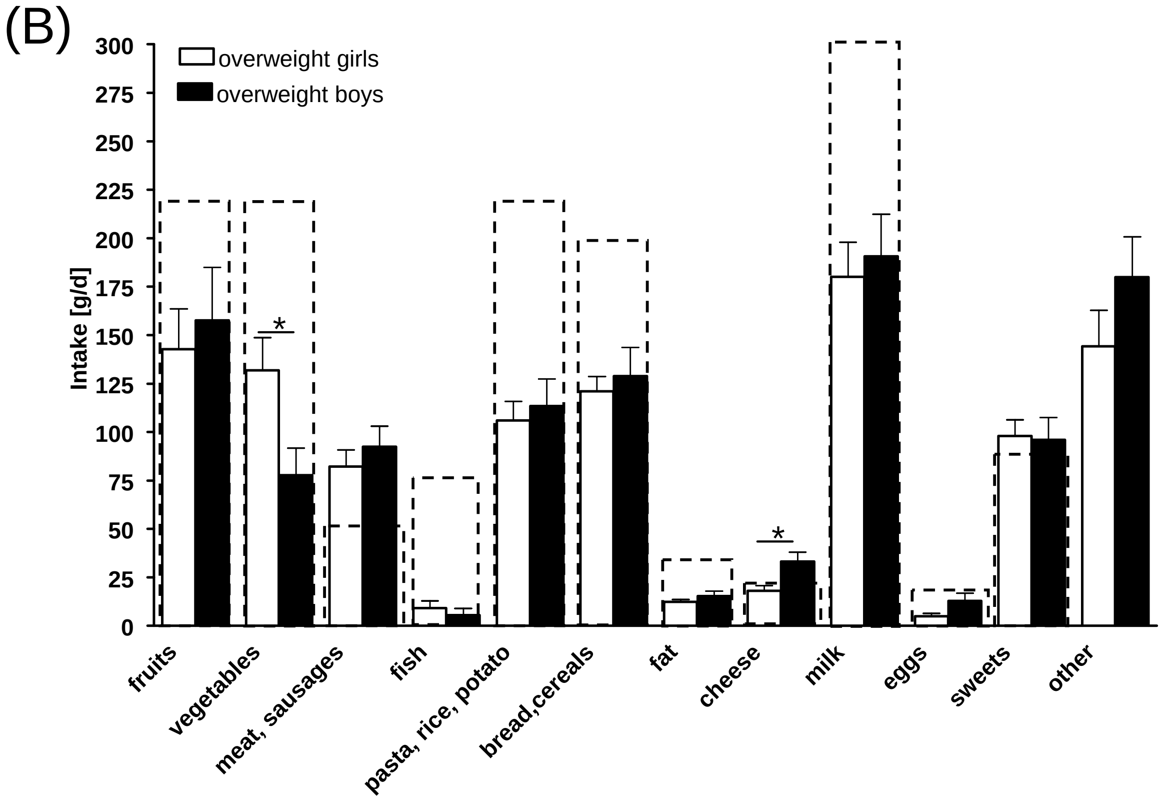

Additional file 1

Intake of different food groups of (A) normal weight girls and normal weight boys and of (B) overweight girls and overweight boys. Recommendations of the German research institute for nutrition of children (FKE) are shown as dashed lines. *p<0.05
